# (*E*)-3-(9-Anthr­yl)-1-(4-fluoro­phen­yl)-2-(1*H*-1,2,4-triazol-1-yl)prop-2-en-1-one

**DOI:** 10.1107/S1600536809039178

**Published:** 2009-10-03

**Authors:** Guang-Zhou Wang, Min Su, Cheng-He Zhou

**Affiliations:** a Laboratory of Bioorganic & Medicinal Chemistry, School of Chemistry and Chemical Engineering, Southwest University, Chongqing 400715, People’s Republic of China; b College of Pharmacy, The Third Military Medical University, Chongqing 400038, People’s Republic of China

## Abstract

The C=C double-bond in the title compound, C_25_H_16_FN_3_O, has an *E* configuration. The dihedral angle between the fluoro­phenyl and triazole rings is 80.57 (2)°.

## Related literature

For the synthesis, see: Erhardt *et al.* (1985 ▶); Kranz *et al.* (1980 ▶). For the pharmacological activity of azoles including imidazole and triazole derivatives, see: Luo *et al.* (2009 ▶); Zhou *et al.* (2009 ▶). For related structures, see: Lu *et al.* (2009 ▶); Wang *et al.* (2009 ▶); Yan *et al.* (2009 ▶).
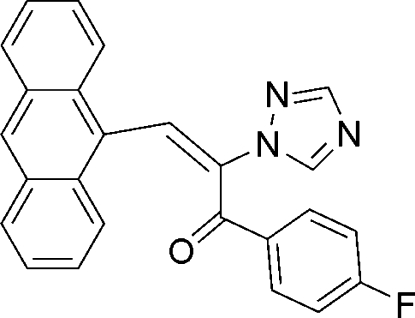


## Experimental

### 

#### Crystal data

C_25_H_16_FN_3_O
*M* *_r_* = 393.41
Orthorhombic, 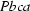

*a* = 13.1115 (8) Å
*b* = 13.4737 (8) Å
*c* = 21.7019 (14) Å
*V* = 3833.9 (4) Å^3^
*Z* = 8
Mo *K*α radiation
μ = 0.09 mm^−1^
*T* = 298 K
0.20 × 0.10 × 0.10 mm

#### Data collection

Bruker SMART diffractometer
Absorption correction: multi-scan (*SADABS*; Sheldrick, 1996 ▶) *T*_min_ = 0.982, *T*_max_ = 0.991
16309 measured reflections
4187 independent reflections
3550 reflections with *I* > 2σ(*I*)
*R*_int_ = 0.041

#### Refinement

*R*[*F* ^2^ > 2σ(*F* ^2^)] = 0.064
*wR*(*F* ^2^) = 0.152
*S* = 1.14
4187 reflections
271 parameters
H-atom parameters constrained
Δρ_max_ = 0.24 e Å^−3^
Δρ_min_ = −0.19 e Å^−3^

### 

Data collection: *SMART* (Bruker, 2001 ▶); cell refinement: *SAINT-Plus* (Bruker, 2001 ▶); data reduction: *SAINT-Plus*; program(s) used to solve structure: *SHELXS97* (Sheldrick, 2008 ▶); program(s) used to refine structure: *SHELXL97* (Sheldrick, 2008 ▶); molecular graphics: *SHELXTL* (Sheldrick, 2008 ▶); software used to prepare material for publication: *SHELXTL*.

## Supplementary Material

Crystal structure: contains datablocks I, global. DOI: 10.1107/S1600536809039178/ng2652sup1.cif

Structure factors: contains datablocks I. DOI: 10.1107/S1600536809039178/ng2652Isup2.hkl

Additional supplementary materials:  crystallographic information; 3D view; checkCIF report

## Figures and Tables

**Fig. 1.**
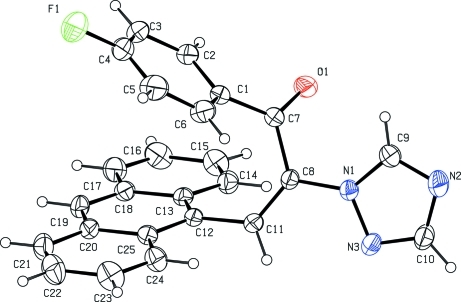
The molecular structure of (I), showing 50% probability displacement ellipsoids and the atom-numbering scheme.

**Fig. 2.**
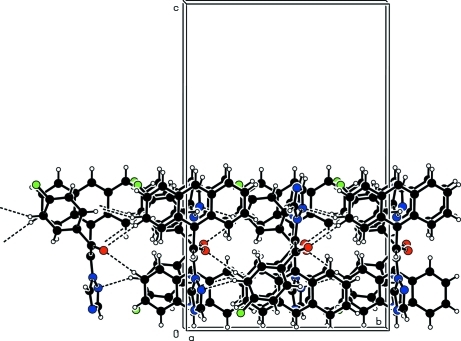
A partial packing diagram. Hydrogen bonds are shown as dashed lines.

## supplementary crystallographic information

### Comment

Chalcones have been investigated for a long time due to important biological activities. We have already synthesized and reported several related structures of chalcones (Lu *et al.*, 2009; Wang *et al.*, 2009; Yan *et al.*, 2009). Azole compounds including imidazole and triazole derivatives are important types of antimicrobial drugs in clinical use, and are excellent ligands with several N-atoms which could coordinate with many kinds of metals (Luo *et al.*, 2009; Zhou *et al.*, 2009). The anthracene moiety is well known for its high absorption co-efficient as well as high fluorescence yields. These interesting properties lead us to develop the title anthryl-triazole chalcone derivatives containing the pharmacophore (triazole) and fluorophore (anthracene), and these compounds exhibit good antimicrobial, antitumor and fluorescent properties. Here we present the title compound (I) crystal structure.

The structure of title compound, C_25_H_16_FN_3_O, has orthorhombic (*Pbca*) symmetry. It is of interest with respect to biological activity. In the structure, the dihedral angle between the benzene and triazole ring is 80.57 (2)°. Weak intermolecular C—H···O and C—H···N interactions contribute to the crystal packing.

### Experimental

Compound (I) was synthesized according to the procedure of Erhardt *et al.* (1985) and Kranz *et al.* (1980). Single crystals used in X-ray diffraction studies were grown in dichlormethane by slow evaporation at room temperature.

### Refinement

All the hydrogen atoms were placed at their geometrical positions with C—H = 0.93Å and *U*_iso_(H) = 1.2*U*_eq_(C).

### Crystal data

**Table:**

C_25_H_16_FN_3_O	*F*(000) = 1632
*M**_r_* = 393.41	*D*_x_ = 1.363 Mg m^−^^3^
Orthorhombic, *P**b**c**a*	Mo *K*α radiation, λ = 0.71073 Å
Hall symbol: -P 2ac 2ab	Cell parameters from 5206 reflections
*a* = 13.1115 (8) Å	θ = 2.4–27.6°
*b* = 13.4737 (8) Å	µ = 0.09 mm^−^^1^
*c* = 21.7019 (14) Å	*T* = 298 K
*V* = 3833.9 (4) Å^3^	Block, yellow
*Z* = 8	0.20 × 0.10 × 0.10 mm

### Data collection

**Table:**

Bruker SMART diffractometer	4187 independent reflections
Radiation source: fine-focus sealed tube	3550 reflections with *I* > 2σ(*I*)
graphite	*R*_int_ = 0.041
φ and ω scans	θ_max_ = 27.0°, θ_min_ = 2.4°
Absorption correction: multi-scan (*SADABS*; Sheldrick, 1996)	*h* = −16→6
*T*_min_ = 0.982, *T*_max_ = 0.991	*k* = −12→17
16309 measured reflections	*l* = −27→26

### Refinement

**Table:**

Refinement on *F*^2^	Primary atom site location: structure-invariant direct methods
Least-squares matrix: full	Secondary atom site location: difference Fourier map
*R*[*F*^2^ > 2σ(*F*^2^)] = 0.064	Hydrogen site location: inferred from neighbouring sites
*wR*(*F*^2^) = 0.152	H-atom parameters constrained
*S* = 1.14	*w* = 1/[σ^2^(*F*_o_^2^) + (0.0581*P*)^2^ + 1.3538*P*] where *P* = (*F*_o_^2^ + 2*F*_c_^2^)/3
4187 reflections	(Δ/σ)_max_ = 0.001
271 parameters	Δρ_max_ = 0.24 e Å^−^^3^
0 restraints	Δρ_min_ = −0.19 e Å^−^^3^

### Special details

**Table:**

Geometry. All e.s.d.'s (except the e.s.d. in the dihedral angle between two l.s. planes) are estimated using the full covariance matrix. The cell e.s.d.'s are taken into account individually in the estimation of e.s.d.'s in distances, angles and torsion angles; correlations between e.s.d.'s in cell parameters are only used when they are defined by crystal symmetry. An approximate (isotropic) treatment of cell e.s.d.'s is used for estimating e.s.d.'s involving l.s. planes.
Refinement. Refinement of *F*^2^ against ALL reflections. The weighted *R*-factor *wR* and goodness of fit *S* are based on *F*^2^, conventional *R*-factors *R* are based on *F*, with *F* set to zero for negative *F*^2^. The threshold expression of *F*^2^ > σ(*F*^2^) is used only for calculating *R*-factors(gt) *etc*. and is not relevant to the choice of reflections for refinement. *R*-factors based on *F*^2^ are statistically about twice as large as those based on *F*, and *R*- factors based on ALL data will be even larger.

### Fractional atomic coordinates and isotropic or equivalent isotropic displacement parameters (Å^2^)

**Table:**

	*x*	*y*	*z*	*U*_iso_*/*U*_eq_	
C1	0.33997 (13)	−0.03462 (14)	0.19230 (8)	0.0395 (4)	
C2	0.28391 (14)	−0.00708 (17)	0.14089 (9)	0.0471 (5)	
H2	0.2586	0.0572	0.1377	0.057*	
C3	0.26557 (17)	−0.0751 (2)	0.09435 (10)	0.0613 (6)	
H3	0.2296	−0.0569	0.0592	0.074*	
C4	0.30156 (18)	−0.1692 (2)	0.10137 (12)	0.0676 (7)	
C5	0.35496 (19)	−0.19944 (19)	0.15162 (13)	0.0710 (7)	
H5	0.3771	−0.2648	0.1551	0.085*	
C6	0.37565 (16)	−0.13104 (16)	0.19739 (11)	0.0544 (5)	
H6	0.4136	−0.1498	0.2317	0.065*	
C7	0.35581 (14)	0.03737 (14)	0.24300 (8)	0.0385 (4)	
C8	0.45641 (13)	0.03523 (13)	0.27672 (8)	0.0360 (4)	
C9	0.37480 (17)	0.02786 (19)	0.38130 (9)	0.0587 (6)	
H9	0.3100	0.0069	0.3697	0.070*	
C10	0.50043 (18)	0.07070 (17)	0.43119 (9)	0.0542 (5)	
H10	0.5415	0.0867	0.4647	0.065*	
C11	0.54642 (14)	0.03428 (14)	0.24860 (8)	0.0394 (4)	
H11	0.6044	0.0310	0.2732	0.047*	
C12	0.56121 (13)	0.03800 (14)	0.18104 (8)	0.0376 (4)	
C13	0.53007 (13)	0.12230 (14)	0.14735 (8)	0.0392 (4)	
C14	0.48685 (16)	0.20863 (15)	0.17516 (9)	0.0475 (5)	
H14	0.4783	0.2104	0.2177	0.057*	
C15	0.45807 (18)	0.28783 (18)	0.14105 (11)	0.0589 (6)	
H15	0.4301	0.3430	0.1605	0.071*	
C16	0.46980 (19)	0.28793 (19)	0.07665 (11)	0.0634 (6)	
H16	0.4485	0.3424	0.0537	0.076*	
C17	0.51180 (17)	0.20924 (18)	0.04815 (10)	0.0576 (6)	
H17	0.5198	0.2105	0.0056	0.069*	
C18	0.54443 (14)	0.12368 (16)	0.08176 (8)	0.0437 (5)	
C19	0.58943 (16)	0.04302 (16)	0.05307 (9)	0.0488 (5)	
H19	0.5968	0.0439	0.0105	0.059*	
C20	0.62388 (14)	−0.03901 (15)	0.08563 (9)	0.0437 (5)	
C21	0.67370 (18)	−0.12021 (18)	0.05638 (10)	0.0583 (6)	
H21	0.6821	−0.1195	0.0138	0.070*	
C22	0.7087 (2)	−0.19767 (19)	0.08895 (12)	0.0696 (7)	
H22	0.7412	−0.2498	0.0689	0.083*	
C23	0.6965 (2)	−0.20059 (18)	0.15352 (12)	0.0667 (7)	
H23	0.7214	−0.2545	0.1757	0.080*	
C24	0.64870 (17)	−0.12541 (16)	0.18361 (10)	0.0532 (5)	
H24	0.6408	−0.1288	0.2261	0.064*	
C25	0.61067 (14)	−0.04183 (14)	0.15128 (8)	0.0405 (4)	
F1	0.28332 (14)	−0.23601 (15)	0.05633 (9)	0.1089 (7)	
N1	0.45059 (11)	0.04595 (11)	0.34187 (7)	0.0386 (4)	
N2	0.40276 (16)	0.04324 (16)	0.43811 (8)	0.0624 (5)	
N3	0.53431 (13)	0.07357 (14)	0.37481 (7)	0.0507 (4)	
O1	0.29069 (10)	0.09647 (12)	0.25734 (7)	0.0580 (4)	

### Atomic displacement parameters (Å^2^)

**Table:**

	*U*^11^	*U*^22^	*U*^33^	*U*^12^	*U*^13^	*U*^23^
C1	0.0319 (9)	0.0475 (11)	0.0392 (9)	−0.0033 (8)	0.0027 (7)	−0.0032 (8)
C2	0.0384 (10)	0.0588 (12)	0.0442 (11)	−0.0026 (9)	−0.0005 (8)	−0.0006 (9)
C3	0.0460 (11)	0.0943 (19)	0.0437 (11)	−0.0103 (12)	−0.0051 (9)	−0.0090 (12)
C4	0.0497 (12)	0.0877 (19)	0.0653 (15)	−0.0100 (13)	0.0023 (11)	−0.0383 (14)
C5	0.0600 (14)	0.0563 (14)	0.097 (2)	0.0019 (12)	−0.0040 (14)	−0.0270 (14)
C6	0.0499 (12)	0.0502 (12)	0.0632 (13)	0.0028 (10)	−0.0088 (10)	−0.0064 (10)
C7	0.0342 (9)	0.0441 (10)	0.0372 (9)	−0.0024 (8)	0.0023 (7)	−0.0008 (8)
C8	0.0386 (9)	0.0398 (10)	0.0297 (8)	−0.0014 (7)	−0.0009 (7)	−0.0012 (7)
C9	0.0463 (11)	0.0879 (17)	0.0418 (11)	−0.0146 (11)	0.0088 (9)	0.0002 (11)
C10	0.0641 (13)	0.0643 (14)	0.0343 (10)	−0.0054 (11)	−0.0020 (9)	−0.0053 (9)
C11	0.0350 (9)	0.0499 (11)	0.0334 (9)	−0.0006 (8)	−0.0030 (7)	0.0003 (8)
C12	0.0307 (8)	0.0506 (11)	0.0315 (9)	−0.0065 (8)	0.0011 (7)	−0.0026 (8)
C13	0.0324 (8)	0.0494 (11)	0.0358 (9)	−0.0089 (8)	−0.0008 (7)	−0.0002 (8)
C14	0.0499 (11)	0.0516 (12)	0.0411 (10)	−0.0046 (9)	0.0025 (9)	−0.0013 (9)
C15	0.0624 (14)	0.0503 (13)	0.0641 (14)	0.0032 (10)	0.0060 (11)	0.0028 (11)
C16	0.0622 (14)	0.0642 (15)	0.0637 (14)	0.0088 (12)	0.0056 (11)	0.0229 (12)
C17	0.0559 (13)	0.0763 (16)	0.0405 (11)	−0.0025 (12)	0.0027 (9)	0.0155 (11)
C18	0.0378 (9)	0.0573 (12)	0.0360 (10)	−0.0068 (9)	0.0002 (8)	0.0032 (9)
C19	0.0485 (11)	0.0691 (14)	0.0288 (9)	−0.0086 (10)	0.0025 (8)	−0.0007 (9)
C20	0.0389 (10)	0.0542 (12)	0.0381 (10)	−0.0091 (8)	0.0022 (8)	−0.0093 (9)
C21	0.0662 (14)	0.0628 (15)	0.0460 (12)	−0.0052 (12)	0.0095 (10)	−0.0147 (11)
C22	0.0804 (17)	0.0574 (15)	0.0710 (16)	0.0056 (13)	0.0183 (13)	−0.0151 (13)
C23	0.0755 (16)	0.0506 (13)	0.0741 (16)	0.0068 (12)	0.0109 (13)	0.0064 (12)
C24	0.0554 (12)	0.0562 (13)	0.0481 (12)	0.0005 (10)	0.0085 (10)	0.0039 (10)
C25	0.0355 (9)	0.0472 (11)	0.0389 (10)	−0.0084 (8)	0.0027 (7)	−0.0014 (8)
F1	0.0917 (12)	0.1308 (15)	0.1043 (13)	−0.0117 (11)	−0.0058 (10)	−0.0780 (12)
N1	0.0392 (8)	0.0445 (9)	0.0321 (8)	−0.0036 (7)	0.0021 (6)	−0.0009 (6)
N2	0.0652 (12)	0.0856 (14)	0.0365 (9)	−0.0080 (11)	0.0098 (8)	0.0014 (9)
N3	0.0491 (9)	0.0681 (12)	0.0350 (8)	−0.0111 (8)	−0.0023 (7)	−0.0072 (8)
O1	0.0419 (8)	0.0665 (10)	0.0657 (10)	0.0116 (7)	−0.0041 (7)	−0.0224 (8)

### Geometric parameters (Å, °)

**Table:**

C1—C6	1.385 (3)	C12—C25	1.412 (3)
C1—C2	1.387 (3)	C13—C14	1.428 (3)
C1—C7	1.481 (3)	C13—C18	1.436 (3)
C2—C3	1.385 (3)	C14—C15	1.352 (3)
C2—H2	0.9300	C14—H14	0.9300
C3—C4	1.362 (4)	C15—C16	1.406 (3)
C3—H3	0.9300	C15—H15	0.9300
C4—F1	1.350 (3)	C16—C17	1.345 (3)
C4—C5	1.358 (4)	C16—H16	0.9300
C5—C6	1.382 (3)	C17—C18	1.430 (3)
C5—H5	0.9300	C17—H17	0.9300
C6—H6	0.9300	C18—C19	1.385 (3)
C7—O1	1.208 (2)	C19—C20	1.387 (3)
C7—C8	1.509 (2)	C19—H19	0.9300
C8—C11	1.329 (2)	C20—C21	1.424 (3)
C8—N1	1.423 (2)	C20—C25	1.436 (3)
C9—N2	1.303 (3)	C21—C22	1.342 (3)
C9—N1	1.334 (2)	C21—H21	0.9300
C9—H9	0.9300	C22—C23	1.411 (4)
C10—N3	1.302 (3)	C22—H22	0.9300
C10—N2	1.341 (3)	C23—C24	1.358 (3)
C10—H10	0.9300	C23—H23	0.9300
C11—C12	1.480 (2)	C24—C25	1.417 (3)
C11—H11	0.9300	C24—H24	0.9300
C12—C13	1.411 (3)	N1—N3	1.362 (2)
			
C6—C1—C2	119.61 (18)	C15—C14—H14	119.3
C6—C1—C7	120.50 (17)	C13—C14—H14	119.3
C2—C1—C7	119.78 (18)	C14—C15—C16	120.9 (2)
C3—C2—C1	120.1 (2)	C14—C15—H15	119.5
C3—C2—H2	119.9	C16—C15—H15	119.5
C1—C2—H2	119.9	C17—C16—C15	120.1 (2)
C4—C3—C2	118.3 (2)	C17—C16—H16	120.0
C4—C3—H3	120.8	C15—C16—H16	120.0
C2—C3—H3	120.8	C16—C17—C18	121.6 (2)
F1—C4—C5	118.2 (3)	C16—C17—H17	119.2
F1—C4—C3	118.6 (3)	C18—C17—H17	119.2
C5—C4—C3	123.2 (2)	C19—C18—C17	122.07 (18)
C4—C5—C6	118.6 (2)	C19—C18—C13	119.44 (18)
C4—C5—H5	120.7	C17—C18—C13	118.49 (19)
C6—C5—H5	120.7	C18—C19—C20	122.34 (17)
C5—C6—C1	120.1 (2)	C18—C19—H19	118.8
C5—C6—H6	119.9	C20—C19—H19	118.8
C1—C6—H6	119.9	C19—C20—C21	122.31 (19)
O1—C7—C1	121.59 (17)	C19—C20—C25	119.16 (18)
O1—C7—C8	120.36 (17)	C21—C20—C25	118.53 (19)
C1—C7—C8	118.06 (16)	C22—C21—C20	121.3 (2)
C11—C8—N1	120.33 (16)	C22—C21—H21	119.3
C11—C8—C7	123.64 (16)	C20—C21—H21	119.3
N1—C8—C7	115.66 (15)	C21—C22—C23	120.4 (2)
N2—C9—N1	111.61 (19)	C21—C22—H22	119.8
N2—C9—H9	124.2	C23—C22—H22	119.8
N1—C9—H9	124.2	C24—C23—C22	120.6 (2)
N3—C10—N2	116.04 (19)	C24—C23—H23	119.7
N3—C10—H10	122.0	C22—C23—H23	119.7
N2—C10—H10	122.0	C23—C24—C25	121.1 (2)
C8—C11—C12	124.83 (16)	C23—C24—H24	119.4
C8—C11—H11	117.6	C25—C24—H24	119.4
C12—C11—H11	117.6	C12—C25—C24	122.70 (17)
C13—C12—C25	120.59 (16)	C12—C25—C20	119.28 (17)
C13—C12—C11	120.18 (17)	C24—C25—C20	118.02 (18)
C25—C12—C11	119.17 (17)	C9—N1—N3	108.28 (15)
C12—C13—C14	123.48 (17)	C9—N1—C8	131.20 (16)
C12—C13—C18	119.09 (17)	N3—N1—C8	120.40 (14)
C14—C13—C18	117.41 (17)	C9—N2—C10	101.93 (17)
C15—C14—C13	121.49 (19)	C10—N3—N1	102.13 (16)
			
C6—C1—C2—C3	−1.3 (3)	C14—C13—C18—C19	177.72 (18)
C7—C1—C2—C3	−177.61 (18)	C12—C13—C18—C17	179.52 (17)
C1—C2—C3—C4	1.7 (3)	C14—C13—C18—C17	−2.0 (3)
C2—C3—C4—F1	179.6 (2)	C17—C18—C19—C20	178.12 (19)
C2—C3—C4—C5	−0.3 (4)	C13—C18—C19—C20	−1.6 (3)
F1—C4—C5—C6	178.8 (2)	C18—C19—C20—C21	−177.50 (19)
C3—C4—C5—C6	−1.3 (4)	C18—C19—C20—C25	1.4 (3)
C4—C5—C6—C1	1.6 (4)	C19—C20—C21—C22	178.3 (2)
C2—C1—C6—C5	−0.4 (3)	C25—C20—C21—C22	−0.6 (3)
C7—C1—C6—C5	175.9 (2)	C20—C21—C22—C23	0.2 (4)
C6—C1—C7—O1	−141.0 (2)	C21—C22—C23—C24	0.3 (4)
C2—C1—C7—O1	35.2 (3)	C22—C23—C24—C25	−0.5 (4)
C6—C1—C7—C8	39.1 (2)	C13—C12—C25—C24	176.20 (17)
C2—C1—C7—C8	−144.71 (18)	C11—C12—C25—C24	−1.1 (3)
O1—C7—C8—C11	−131.1 (2)	C13—C12—C25—C20	−3.5 (3)
C1—C7—C8—C11	48.8 (3)	C11—C12—C25—C20	179.16 (16)
O1—C7—C8—N1	41.8 (3)	C23—C24—C25—C12	−179.6 (2)
C1—C7—C8—N1	−138.21 (17)	C23—C24—C25—C20	0.2 (3)
N1—C8—C11—C12	−170.82 (17)	C19—C20—C25—C12	1.2 (3)
C7—C8—C11—C12	1.8 (3)	C21—C20—C25—C12	−179.87 (18)
C8—C11—C12—C13	63.7 (3)	C19—C20—C25—C24	−178.53 (18)
C8—C11—C12—C25	−118.9 (2)	C21—C20—C25—C24	0.4 (3)
C25—C12—C13—C14	−175.05 (17)	N2—C9—N1—N3	1.0 (3)
C11—C12—C13—C14	2.2 (3)	N2—C9—N1—C8	176.88 (19)
C25—C12—C13—C18	3.3 (3)	C11—C8—N1—C9	−163.3 (2)
C11—C12—C13—C18	−179.43 (16)	C7—C8—N1—C9	23.5 (3)
C12—C13—C14—C15	179.98 (19)	C11—C8—N1—N3	12.2 (3)
C18—C13—C14—C15	1.6 (3)	C7—C8—N1—N3	−160.99 (17)
C13—C14—C15—C16	0.0 (3)	N1—C9—N2—C10	−0.7 (3)
C14—C15—C16—C17	−1.2 (4)	N3—C10—N2—C9	0.2 (3)
C15—C16—C17—C18	0.7 (4)	N2—C10—N3—N1	0.4 (3)
C16—C17—C18—C19	−178.8 (2)	C9—N1—N3—C10	−0.8 (2)
C16—C17—C18—C13	0.9 (3)	C8—N1—N3—C10	−177.23 (17)
C12—C13—C18—C19	−0.7 (3)		

### Hydrogen-bond geometry (Å, °)

**Table:**

*D*—H···*A*	*D*—H	H···*A*	*D*···*A*	*D*—H···*A*
C23—H23···O1^i^	0.93	2.48	3.354 (3)	156
C5—H5···N3^i^	0.93	2.55	3.434 (3)	158

Symmetry codes: (i) −*x*+1, *y*−1/2, −*z*+1/2.
